# Ratio of Pro-Resolving and Pro-Inflammatory Lipid Mediator Precursors as Potential Markers for Aggressive Periodontitis

**DOI:** 10.1371/journal.pone.0070838

**Published:** 2013-08-12

**Authors:** Hager R. Zein Elabdeen, Manal Mustafa, Monika Szklenar, Ralph Rühl, Raouf Ali, Anne Isine Bolstad

**Affiliations:** 1 Department of Clinical Dentistry – Periodontics, University of Bergen, Bergen, Norway; 2 Paprika Bioanalytics Bt., Debrecen, Hungary; 3 Department of Biochemistry and Molecular Biology, University of Debrecen, Debrecen, Hungary; 4 Faculty of Dentistry, University of Science and Technology, Omdurman, Sudan; Albert Einstein College of Medicine, United States of America

## Abstract

Aggressive periodontitis (AgP) is a rapidly progressing type of periodontal disease in otherwise healthy individuals which causes destruction of the supporting tissues of the teeth. The disease is initiated by pathogenic bacteria in the dental biofilm, and the severity of inflammation and attachment loss varies with the host response. Recently, there has been an increased interest in determining the role of lipid mediators in inflammatory events and the concept of pro-inflammatory and pro-resolution lipid mediators has been brought into focus also in periodontal disease. The present study aimed to determine the profile of omega-3 or n3- as well as omega-6 or n6- polyunsaturated fatty acids (PUFAs) and PUFA-metabolites of linoleic acid, arachidonic acid (AA), eicosapentaenoic acid (EPA) and docosahexaenoic acid (DHA) in gingival crevicular fluid (GCF), saliva and serum in AgP patients and healthy controls. In total, 60 selected n3- and n6-PUFAs and various PUFA metabolites were measured using high performance liquid chromatography-tandem electrospray ionisation mass spectrometry (HPLC-ESI-MS-MS). Of these, 51 could be quantified in this study. The concentrations of the majority were low in saliva samples compared with serum and GCF, but were mainly higher in AgP patients compared with healthy controls in all three kinds of sample. Ratios of n3- to n6-PUFAs (DHA + EPA)/AA were significantly lower in the GCF of AgP patients than in the healthy controls. Furthermore, various ratios of the direct precursors of the pro-resolution lipid mediators (precursors of resolvins and protectins) were calculated against the precursors of mainly pro-inflammatory lipid mediators. These ratios were mainly lower in GCF and saliva of AgP patients, compared with healthy controls, but only reached significance in GCF (P<0.05). To conclude, the ratios of precursors of pro-resolution/pro-inflammatory lipid mediators seem to be more relevant for describing the disease status of AgP than the concentration of specific lipid mediators.

## Introduction

Aggressive periodontitis (AgP) is a rapidly progressive form of inflammatory periodontal disease. If not treated, AgP may result in tooth loss even in young people [1]. The initiating factors of periodontal disease are bacteria and bacterial products which form a biofilm covering the tooth surface in the subgingival area. In susceptible individuals, the host-mediated response to the pathogenic flora is subsequently responsible for the periodontal tissue destruction [2], [3]. The course of the disease is modified by different environmental factors, and the patient's genetic make-up contributes to patient susceptibility, in particular in the case of aggressive forms of periodontitis [4]–[6].

Persistence of inflammation or the failure of tissue to return to the normal state favours tissue destruction [7]. Thus, resolution of inflammation is essential to the process of regaining tissue homeostasis after infection, and it has become clear that this is not a passively, but an actively coordinated process involving several biochemical pathways, enzymes and mediators [8], [9]. Over recent years, there has been an increased interest in determining the role of lipid mediators in anti-inflammatory events, and the concept of pro-inflammatory and pro-resolution lipid mediators has also been brought into focus in periodontal disease pathogenesis [10].

Host factors, including both immune components and resident cells, contribute to tissue destruction via synthesis and release of different pro-inflammatory molecules [11]. The pathogens trigger the cell membrane of the immune cells, leading to the release of free fatty acid, mainly arachidonic acid (AA). Pro-inflammatory lipid mediators such as thromboxanes (TX), prostaglandins (PG) and leukotrienes (LT), as well as anti-inflammatory lipid mediators such as lipoxins (LX), are among the products generated, mainly via AA-metabolism.

Arachidonic acid is metabolized to biologically active eicosanoids through the cyclooxygenase pathway (COX-1 and COX-2), which results in production of various PG-like PGE_2_ and PGD_2_ [12], [13]. Other mediators such as LT, particularly LTB_4,_ arise through the conversion of hydroxyeicosatetraenoic acid (HETE) by 5-lipoxygenase (5-LOX). The AA-derived prostanoids and LT are associated with the destruction of collagen and bone resorption occurring in periodontal disease [14], [15]. Lipoxygenases involved in AA, EPA and DHA metabolism are the 5-LOX, 12-LOX and 15-LOX. The human 12-LOX can be classified into two types, the platelet type and the epidermis type [16], [17] while the human type can be classified into the reticulocyte type (15-LOX-1) [18], leukocyte type [19], and the epidermis type (15-LOX-2) [20], [21]. Prostaglandins are biological mediators involved in inflammation and have been implicated as stimulators of bone loss [8]. Levels of PGE_2_ are significantly elevated in the gingival crevicular fluid (GCF) of patients with periodontal disease. High levels of PGE_2_ are associated with disease aggressiveness and constitute a reliable indicator of current clinical periodontal destruction [14], [15]. High levels of LTB_4_ have also been found in periodontal inflamed tissues and are considered to be an indicator of periodontal inflammation [14].

**Figure 1:**
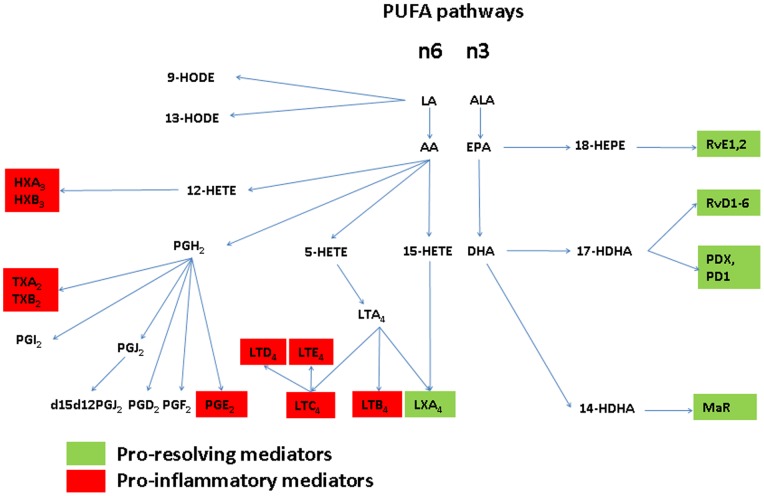
PUFA-metabolites and lipid mediators originating from the n6- and n3-polyunsaturated fatty acids (PUFAs). In addition, bioactive pro-resolving as well as pro-inflammatory eicosanoids and docosanoids were marked in red or green, respectively. Abbreviations: AA: arachidonic acid; ALA: alpha-linolenic; DHA: docosahexaenoic acid, EPA: eicosapentaenoic acid; HEPE: hydroxyeicosapentaenoic acid; HETE: hydroxyeicosatetraenoic acid; HDHA: hydroxydocosahexaenoic acid¸ LA: linoleic acid; LT: leukotriene; LX: lipoxin, HODE: hydroxyoctadecadienoic acid; HX: hepoxilin; MaR: maresin; PD: protectin; PG: prostaglandin; Rv: resolvin; TX: thromboxane.

Omega-6 (n6) and omega-3 (n3) polyunsaturated fatty acids (PUFAs) are substrates for the production of various eicosanoids and docosanoids (Figure 1). The mono-hydroxylated PUFA metabolites of AA, 15-HETE and 5-HETE are direct precursors for LXs, which, in contrast to PGs and LTs, attenuate the inflammatory effect [22], [23]. The major n3-PUFAs are eicosapentaenoic acid (EPA) and docosahexaenoic acid (DHA), which are the precursors of various novel pro-resolution lipid mediators like resolvins (Rv), protectins (PD) and maresins (MaR) which are responsible for the active resolution of inflammation [24]–[27]. The PUFAs EPA and DHA are substrates for various classes of Rv of the E (originating from EPA) and D (originating from DHA) series, respectively [28]. Interestingly, local application of RvE1, which is one of the bioactive pro-resolution mediators, reduced inflammation and promoted regeneration of pathologically lost tissues in a rabbit model of periodontal disease [29]. In humans, the daily dietary supplementation of n3-PUFAs in combination with aspirin was reported to reduce the frequency of deep periodontal pockets and inflammatory lipid mediators in saliva [30].

Little is known about the presence and the amount of different bioactive lipid mediators in oral fluids during periodontal inflammation and its resolution. In particular, a comparison between their levels locally near the inflamed site (in GCF and saliva) and systemically (in serum) has, to our knowledge, not been done. In this report, we analysed eicosanoids and docosanoids in GCF, saliva and serum in a group of AgP patients and healthy controls in an attempt to understand better the roles and relationships of these lipid mediators.

## Results

In the present study the levels of various PUFAs and mono-hydroxylated PUFA metabolites and pro-resolution and pro-inflammatory lipid mediators in GCF, saliva and serum samples from AgP patients and healthy controls were measured by high performance liquid chromatography – electrospray ionization combined with tandem mass spectrometry (HPLC-ESI-MS-MS) and enzyme-linked immunosorbent assay (ELISA). Not all samples could be analysed, and the final numbers of individuals analysed for each sample type are specified in Tables 1, 2 and 3. In total for each individual, 60 lipids were measured using the HPLC-ESI-MS-MS method and 51 could be quantified in the biological liquids. The concentrations of these lipids were lower in saliva samples (Table 2) compared with both serum (Table 3) and GCF samples (Table 1). Levels of these lipids were increased in most biological samples of AgP patients compared with healthy controls, although not all results were statistically significant.

**Table 1:** Concentration of lipid mediators in GCF samples.

		AgP	HC	P value
		(n = 16) ng/ml	(n = 12) ng/ml	
**PUFAs**	AA	15.5±15.5	2.7±2.3	0.001*
	EPA	0.2±0.2	0.1±0.0†	0.012*
	DHA	1.0±0.7	0.4±0.2	0.046*
	LA	17.2±8.6	9.7±4.7	0.007*
**5-lipoxygenase pathway**	5-HETE	0.1±0.1	0.1±0.0†	0.179
	LTB_4_	0.1±0.1	0.1±0.0†	0.113
	20-OH-LTB_4_	0.4±0.5	0.2±0.2	0.690
	20-COOH-LTB_4_	3.1±2.7	2.1±4.5	0.054
	LTC_4_	0.1±0.1	0.1±0.1†	0.780
	5-oxoETE**	0.6±0.6	0.3±0.3†	0.055
	LXB_4_	0.1±0.1	0.1±0.1†	0.960
	LXA_4_	0.2±0.4	0.1±0.1†	0.146
	5-HEPE	0.1±0.1	0.1±0.0†	0.120
	LTB_5_	0.2±0.1	0.1±0.1†	0.646
	LXA_5_	0.2±0.3	0.3±0.3†	0.129
	4-HDHA	0.1±0.0	0.1±0.0†	0.120
**8-hydroxylation pathway**	8-HETE	0.1±0.1	0.1±0.0†	0.037*
	8-HEPE	0.1±0.1	0.1±0.1†	0.258
	10-HDHA	0.1±0.1	0.1±0.1†	0.356
	PD1	0.1±0.1	0.1±0.0†	0.903
**12-lipoxygenase pathway**	12-HETE	1.1±0.9	0.6±0.7†	0.039*
	12-oxoETE**	0.8±1.3	0.9±0.7†	0.200
	HXA_3_	0.7±0.7	0.8±1.1†	0.815
	HXB_3_	0.9±1.1	1.0±0.9	0.655
	12-HEPE	0.1±0.2	0.1±0.0†	0.020*
	14-HDHA	0.1±0.0	0.1±0.1†	0.331
	RvD1	0.3±0.4	0.2±0.1†	0.848
	RvD2	0.8±0.7	0.7±0.4†	0.422
	MaR	0.1±0.1	0.1±0.1†	0.533
	9-HODE	6.3±6.4	2.6±1.6	0.029*
**15-lipoxygenase pathway**	15-HETE	1.5±2.0	0.1±0.1†	0.001*
	15-oxoETE**	1.6±1.8	0.2±0.2†	0.050
	15-HEPE	0.2±0.2	0.1±0.0†	0.042*
	17-HDHA	0.2±0.3	0.1±0.0†	0.010*
	13-HODE	14.6±13.0	4.9±3.1	0.009*
	13-oxoODE**	14.4±17.4	14.6±9.2	0.486
**Cyclooxygenase pathway**	PGD_2_	0.9±0.8	1.3±1.0†	0.429
	PGE_2_	2.2±1.6	0.7±0.5	0.008*
	d15d12PGD_2_	0.1±0.1	0.1±0.2†	0.823
	PGJ_2_	0.1±0.1	0.1±0.1†	0.785
	d15d12PGJ_2_	0.1±0.0	0.1±0.0†	0.212
	TXB_2_	0.1±0.1	0.1±0.1†	0.674
	PGF_2_	0.3±0.3	0.2±0.2†	0.571
	PGE_3_	1.8±1.9	1.3±1.3	0.632
**Alternative pathways**	20-HETE	0.3±0.2	0.2±0.2†	0.056
	20-COOH-AA	0.8±0.7	0.3±0.4†	0.044*
	18-HEPE	0.9±1.9	1.2±1.7†	0.118
	20-HDHA	0.2±0.2	0.1±0.1†	0.269
	11-HETE	0.1±0.0	0.1±0.0†	0.020*
**Oxidative stress marker**	8iPGF_2_	0.2±0.2	0.1±0.1†	0.021*
	8iPGF_3_	0.8±0.7	0.7±0.7	0.722

The concentration of lipid mediators in gingival crevicular fluid (GCF) of aggressive periodontitis (AgP) patients and healthy controls (HC) was measured by HPLC-MS-MS. Results in ng/ml are expressed as mean ± SD. * Significantly different (*P*<0.05, Mann Whitney test). †Various values below the quantification limit set to 0.05 ng/ml. ** These derivatives may partly origin from autooxidation.

**Table 2:** Concentration of lipid mediators in saliva samples.

		AgP	HC	P value
		(n = 16) ng/ml	(n = 12) ng/ml	
**PUFAs**	AA	12.24±11.15	7.38±5.22	0.108
	EPA	0.20±0.31	0.07±0.06†	0.102
	DHA	1.19±1.73	0.87±0.60	0.477
	LA	2.98±2.39	2.77±2.53	0.793
**5-lipoxygenase pathway**	5-HETE	0.23±0.27	0.06± 0.08†	0.017*
	LTB_4_	0.47±0.41	0.12±0.15†	0.002*
	20-OH-LTB_4_	0.57±0.93	0.13±0.13†	0.048*
	20-COOH-LTB_4_	0.29±0.30	0.32±0.35†	0.832
	LTC_4_	0.02±0.00	0.02±0.00†	–
	5-oxoETE**	0.06±0.05	0.03±0.02†	0.012*
	LXB_4_	0.02±0.00	0.02±0.00†	–
	LXA_4_	0.05±0.09	0.02±0.01†	0.302
	5-HEPE	0.02±0.00	0.02±0.00†	0.737
	LTB_5_	0.02±0.00	0.02±0.00†	0.364
	LXA_5_	0.02±0.01	0.02±0.00†	0.264
	4-HDHA	0.02±0.00	0.02±0.00†	0.224
**8-hydroxylation pathway**	8-HETE	0.17±0.21	0.16±0.32†	0.913
	8-HEPE	0.04±0.06	0.02±0.01†	0.269
	10-HDHA	0.03±0.01	0.02±0.00†	0.047*
	PD1	0.03±0.02	0.02±0.00†	0.085
**12-lipoxygenase pathway**	12-HETE	3.05±2.49	2.41±3.09†	0.488
	12-oxoETE**	1.11±1.57	1.57±1.38†	0.362
	HXA_3_	0.73±1.14	0.25±0.54†	0.120
	HXB_3_	0.69±2.09	0.28±0.50†	0.436
	12-HEPE	0.12±0.14	0.31±0.95†	0.365
	14-HDHA	0.13±0.13	0.13±0.15†	0.915
	RvD1	0.02±0.00	0.02±0.01†	0.295
	RvD2	0.03±0.02	0.03±0.03†	0.391
	MaR	0.02±0.00	0.02±0.00†	0.284
	9-HODE	0.31±0.35	0.26±0.49	0.759
**15-lipoxygenase pathway**	15-HETE	0.22±0.43	0.03±0.02†	0.004*
	15-oxoETE**	0.12±0.21	0.02±0.02†	0.068
	15-HEPE	0.02±0.00	0.02±0.00†	0.203
	17-HDHA	0.04±0.03	0.02±0.01†	0.036*
	13-HODE	0.43±0.45	0.44±0.72	0.965
	13-oxoODE**	11.65±11.82	14.66±19.76	0.570
**Cyclooxygenase pathway**	PGD_2_	0.11±0.09	0.08±0.10†	0.333
	PGE_2_	0.17±0.26	0.08±0.06†	0.142
	d15d12PGD_2_	0.02±0.00	0.02±0.01†	0.327
	PGJ_2_	0.02±0.01	0.02±0.00†	0.571
	d15d12PGJ_2_	0.03±0.03	0.02±0.00†	0.364
	TXB_2_	0.08±0.10	0.05±0.06†	0.334
	PGF_2_	0.03±0.02	0.02±0.01†	0.032*
	PGE_3_	0.09±0.11	0.08±0.12†	0.764
**Alternative pathways**	20-HETE	0.02±0.00	0.02±0.00†	0.252
	20-COOH-AA	1.76±1.95	0.91±0.65†	0.093
	18-HEPE	0.03±0.02	0.02±0.00†	0.138
	20-HDHA	0.04±0.04	0.02±0.01†	0.261
	11-HETE	0.09±0.15	0.02±0.01†	0.083
**Oxidative stress marker**	8iPGF_2_	0.03±0.04	0.02±0.00†	0.165
	8iPGF_3_	3.93±10.35	0.91±1.58†	0.242

The concentration of lipid mediators in saliva of aggressive periodontitis (AgP) patients and healthy controls (HC) was measured by HPLC-MS-MS. Results in ng/ml are expressed as mean ± SD. * Significantly different (*P*<0.05, Mann Whitney test). †Various values below the quantification limit set to 0.02 ng/ml. ** These derivatives may partly origin from autooxidation.

**Table 3:** Concentration of lipid mediators in serum samples.

		AgP	HC	P value
		(n = 16) ng/ml	(n = 12) ng/ml	
**PUFAs**	AA	855.4±325.0	472.6±261.2	0.002*
	EPA	33.9±33.4	8.2±6.9	0.001*
	DHA	175.7±112.7	82.2±61.0	0.003*
	LA	318.9±210.1	225.4±148.7	0.129
**5-lipoxygenase pathway**	5-HETE	24.1±36.1	0.4±0.2	0.000*
	LTB_4_	0.3±0.3	0.2±0.2†	0.500
	20-OH-LTB_4_	0.5±0.6	0.3±0.3†	0.186
	20-COOH-LTB_4_	1.6±2.2	2.5±2.0	0.111
	LTC_4_	0.1±0.1	0.1±0.1†	0.926
	5-oxoETE**	0.5±0.4	0.2±0.2†	0.028*
	LXB_4_	0.2±0.2	0.5±0.1†	0.550
	LXA_4_	1.1±1.3	0.1±0.2†	0.030*
	5-HEPE	1.0±1.4	0.1±0.1†	0.007*
	LTB_5_	0.1±0.0	0.1±0.1†	0.391
	LXA_5_	0.1±0.1	0.2±0.4	0.938
	4-HDHA	6.3±9.2	0.1±0.0†	0.002*
**8-hydroxylation pathway**	8-HETE	1.6±1.2	0.4±0.3	0.001*
	8-HEPE	0.7±0.6	0.3±0.4†	0.047*
	10-HDHA	0.4±0.3	0.1±0.1†	0.019*
	PD1	0.4±0.5	0.1±0.1†	0.074
**12-lipoxygenase pathway**	12-HETE	259.8±212.7	77.3±76.2	0.005*
	12-oxoETE**	2.8±2.2	2.3±3.0	0.100
	HXA_3_	3.1±2.8	0.9±0.8	0.005*
	HXB_3_	0.7±1.7	0.7±0.7	0.053
	12-HEPE	3.2±3.2	1.4±2.0	0.011*
	14-HDHA	5.0±5.3	1.7±1.6	0.017*
	RvD1	0.1±0.2	0.2±0.3†	0.856
	RvD2	6.2±22.8	0.9±1.9	0.383
	MaR	0.2±0.1	0.1±0.1†	0.134
	9-HODE	4.6±4.1	2.6±4.1	0.008*
**15-lipoxygenase pathway**	15-HETE	5.5±4.4	1.3±1.3	0.001*
	15-oxoETE**	0.9±1.3	0.1±0.1†	0.037*
	15-HEPE	0.3±0.4	0.2±0.1†	0.174
	17-HDHA	0.8±0.8	0.2±0.1†	0.003*
	13-HODE	4.5±4.0	1.6 ±1.0	0.005*
	13-oxoODE**	11.2±9.0	5.0±3.6	0.038*
**Cyclooxygenase pathway**	PGD_2_	2.4±2.1	1.9±1.2	0.626
	PGE_2_	4.8±5.6	1.9±1.3	0.129
	d15d12PGD_2_	2.5±2.1	0.9±0.6†	0.002*
	PGJ_2_	0.2±0.2	0.1±0.1†	0.163
	d15d12PGJ_2_	0.1±0.0	0.1±0.0†	0.975
	TXB_2_	200.5±151.7	138.9±191.9	0.058
	PGF_2_	0.4±0.3	0.3±±0.3†	0.207
	PGE_3_	0.7±0.6	1.5±1.7	0.182
**Alternative pathways**	20-HETE	0.4±0.6	0.4±0.3†	0.756
	20-COOH-AA	5.4±4.7	1.8±1.8†	0.001*
	18-HEPE	0.5±0.3	0.3±0.4†	0.003*
	20-HDHA	0.6±0.6	0.1±0.1†	0.001*
	11-HETE	4.2±3.4	1.2±1.2	0.002*
**Oxidative stress marker**	8iPGF_2_	0.3±0.2	0.2±0.2†	0.218
	8iPGF_3_	0.8±0.9	1.3±1.4	0.371

The concentration of lipid mediators in serum of aggressive periodontitis (AgP) patients and healthy controls (HC) was measured by HPLC-MS-MS. Results in ng/ml are expressed as mean ± SD. * Significantly different (*P*<0.05, Mann Whitney test). †Various values below the quantification limit set to 0.05 ng/ml. ** These derivatives may partly origin from autooxidation.

### GCF analysis

Both n6-PUFAs (linoleic acid (LA) and AA) and n3-PUFAs (EPA and DHA) were detected at significantly higher concentrations in the GCF of AgP patients than in healthy controls (Table 1). Furthermore, the 12-LOX pathway metabolites like 12-HETE, 12-hydroxyeicosapentaenoic acid (12-HEPE), 9-hydroxyoctadecanoic acid (9-HODE) and 14-hydroxydocosahexaenoic acid (14-HDHA); the 15-LOX metabolites 15-HETE, 13-HODE, 15-HEPE and 17-HDHA and various other PUFA metabolites like 11-HETE, 20-COOH-AA and the oxidative stress marker 8iPGF_2_ were all present at enhanced concentrations (significantly different) in AgP patient samples (Table 1). Finally and interestingly, the concentration of the COX-pathway metabolite PGE_2_ was significantly higher in GCF samples of AgP patients (2.2±1.6 ng/ml) compared with healthy controls (0.7±0.5 ng/ml) (*P* = 0.008) (Table 1).

**Figure 2:**
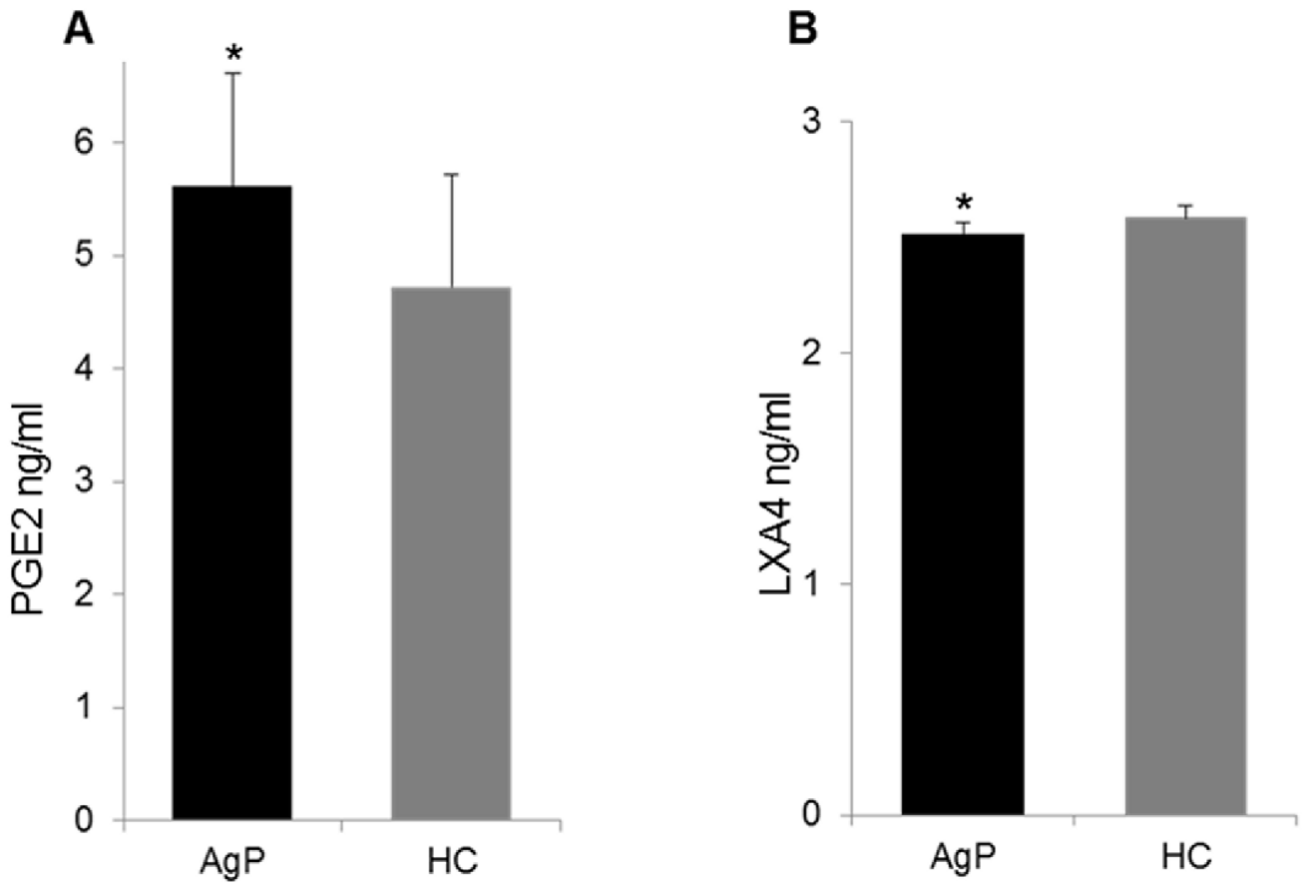
ELISA analysis. Concentration (ng/ml) of PGE_2_ and LXA_4_ as determined by ELISA in gingival crevicular fluid (GCF) samples of aggressive periodontitis (AgP) patients (black bar) and healthy controls (HC) (grey bar) * = *P<*0.05 by use of Mann-Whitney test.

The GCF samples were additionally analysed by ELISA for PGE_2_ and LXA_4._ The levels of PGE_2_ were significantly increased in patients (mean 5.6±1.3 ng/ml) compared with healthy controls (mean 4.7±0.7 ng/ml) (*P* = 0.002), which was in line with the HPLC-ESI-MS-MS results. By use of ELISA, the LXA_4_ concentrations between patients and healthy controls was slightly lower in GCF of AgP compared with healthy controls (*P* = 0.049) (Figure 2).

### Saliva analysis

From the PUFA levels of LA, AA, EPA and DHA were found at slightly higher concentration in saliva samples of AgP patients compared with controls (Table 2). Lipid derivatives originating from the 5-LOX pathways, in particular LTB_4_ (*P* = 0.002), 20-OH-LTB_4_ (*P* = 0.048), 5-HETE (*P* = 0.017), 5-oxoETE and from the 15-LOX pathways, 15-HETE (*P* = 0.004), 15-oxoETE and 17-HDHA, were found in higher concentrations in AgP patients (Table 2). In contrast, 12-oxoETE was present in significantly lower concentrations in the patient group. The concentrations of PGE2 in saliva in AgP patients and healthy controls were not significantly different.

### Serum analysis

As with GCF, the levels of the fatty acids in serum including AA, EPA, DHA and LA, were higher in AgP patients (Table 3). Several PUFA metabolites originating from various LOX pathways, in particular 5-HETE, 8-HETE, 12-HETE, 15-HETE, HXA_3_, 9-HODE, 13-HODE, 13-oxoODE, 5-oxoETE, 15-oxoETE, LXA_4_, 5-HEPE, 12-HEPE, 8-HEPE, 4-HDHA, 10-HDHA, 14-HDHA and 17-HDHA were increased in AgP patient samples compared with healthy controls (Table 3). The concentration of the lipid mediator d15d12PGD2, a COX metabolite, was increased in the serum samples of AgP patients, as well as 20-COOH-AA, 20-HDHA and 11-HETE (Table 3). In general, concentrations of various PUFA metabolites were much higher in serum than in saliva and GCF.

### Ratios of precursors of pro-resolution/pro-inflammatory lipid mediators and LOX/COX-pathway analysis

**Figure 3:**
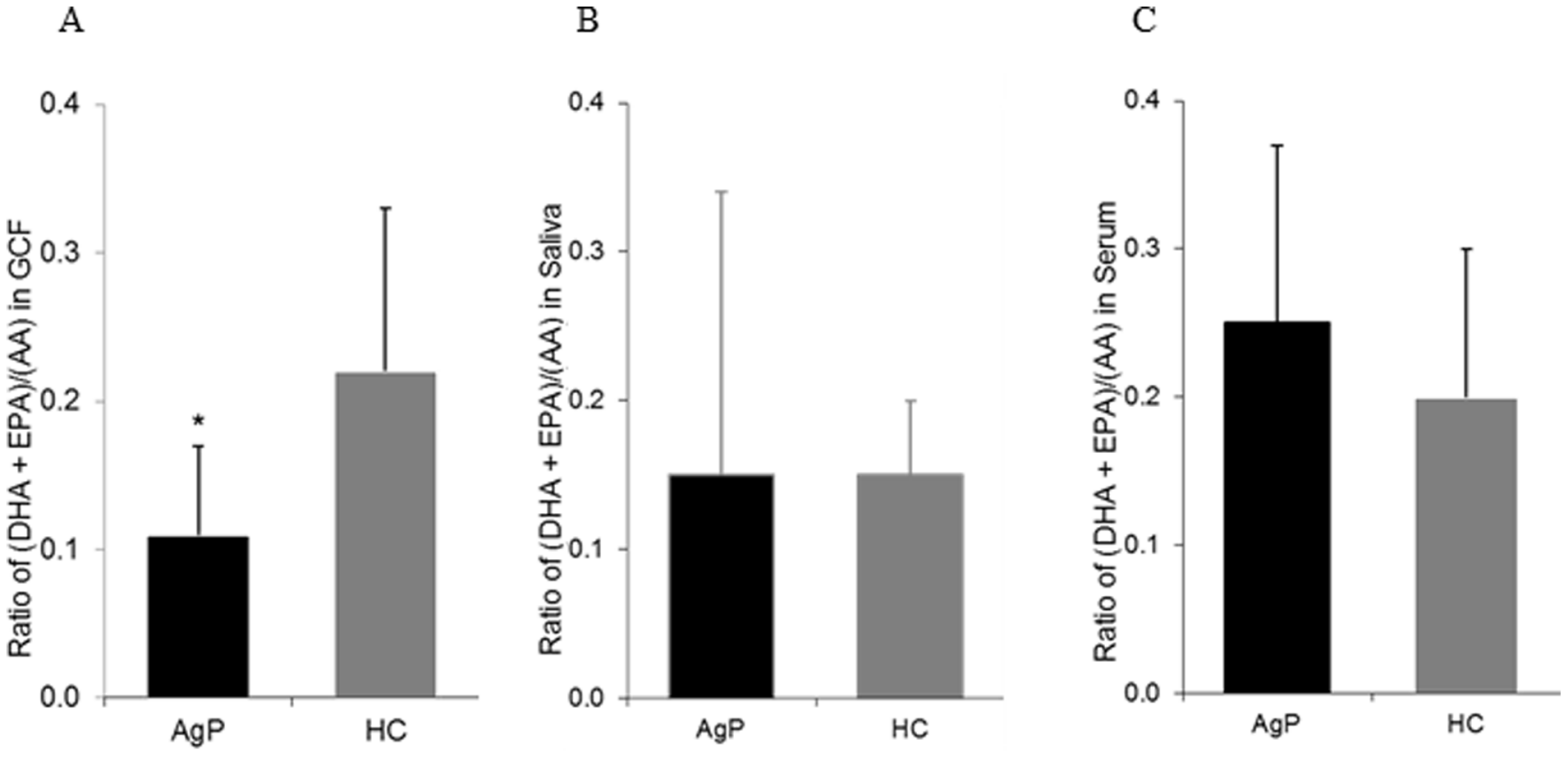
Ratio of n3- to n6-PUFAs in gingival crevicular fluid (GCF) (A), saliva (B) and serum (C) samples. The figure shows the ratios of concentration of (EPA plus DHA) vs. AA in respective fluids of aggressive periodontitis patients (AgP, black bar) and healthy controls (HC, grey bar). * = *P*<0.05 by use of Mann-Whitney test.

There were considerable variations of the concentrations of DHA, EPA and AA between groups in GCF, saliva and serum analysed in this study (Table 1, 2 and 3). The ratio of the n3-PUFAs (DHA and EPA) concentration vs. the concentration the n6-PUFA (AA) in GCF was significantly lower in AgP compared with the control group (*P* = 0.004) (Figure 3A). By contrast, there was no significant difference in these ratios in saliva and serum (Figure 3B and C).

In addition the ratio of the direct precursors of pro-resolution lipid mediators (14-HDHA, 17-HDHA and 18-HEPE), which are precursors of resolvins and protectins, was calculated against various precursors of the AA-derived and mainly pro-inflammatory derivatives (5-HETE, 12-HETE and 15-HETE) as well as ratios of sum of HEPEs and sum of HDHAs vs. the sum of HETEs were calculated (Table 4). The ratio of precursors of pro-resolution to pro-inflammatory lipid markers (HEPE/HETE and HDHA/HETE) were mainly reduced in the GCF, saliva and serum of AgP patients in comparison with healthy controls but was just significant in GCF samples (P<0.05) (Table 4).

**Table 4:** Ratios and sums of lipid mediators from GCF – gingivial crevicular fluid, SAL – saliva, SER – serum of aggressive periodontitis (AgP) patients and healthy controls (HC).

			AgP	HC	P value
			(n = 16)	(n = 12)	
**AA-derived metabolite ratios**	5-HETE/AA	GCF	*0.01±0.01*	0.04±0.03	0.002*
		SAL	**0.04**±**0.07**	0.02±0.03	0.199
		SER	**0.02±0.03**	0.001±0.001	0.010*
	12-HETE/AA	GCF	*0.14±0.16*	0.22±0.13	0.086
		SAL	*0.83±1.75*	1.46±4.25	0.544
		SER	**0.29±0.20**	0.15±0.09	0.062
	15-HETE/AA	GCF	**0.10±0.12**	0.04±0.04	0.178
		SAL	**0.04±0.09**	0.02±0.05	0.303
		SER	**0.01±0.00**	0.003±0.002	0.009*
**EPA-derived metabolite ratios**	18-HEPE/EPA	GCF	**19.67±31.59**	13.90±36.80	0.024*
		SAL	*0.42±0.52*	0.45±0.31	0.802
		SER	*0.03±0.03*	0.06±0.12	0.918
**DHA-derived metabolite ratios**	14-HDHA/DHA	GCF	*0.13±0.11*	0.20±0.10	0.046*
		SAL	*0.38±0.63*	0.58±1.58	0.613
		SER	**0.03±0.02**	0.02±0.02	0.654
	17-HDHA/DHA	GCF	**0.37±0.54**	0.17±0.08	0.754
		SAL	**0.18±0.27**	0.10±0.22	0.345
		SER	**0.004±0.003**	0.003±0.003	0.227
**PUFA-ratios**	EPA/AA	GCF	*0.02±0.01*	0.03±0.02	0.016*
		SAL	0.02±0.02	0.02±0.03	0.805
		SER	**0.03±0.03**	0.02±0.02	0.512
	DHA/AA	GCF	*0.09±0.06*	0.18±0.10	0.008*
		SAL	0.13*±*0.17	0.13±0.04	0.982
		SER	**0.21±0.90**	0.19±0.09	0.629
**Sum of PUFA-originating metabolites**	SUM HETEs	GCF	**3.13±2.56**	1.00±0.82	0.003*
		SAL	**3.76±2.85**	2.69±3.20	0.288
		SER	**260±236**	195±208	0.002*
	SUM HEPEs	GCF	*1.38±1.91*	1.41±1.66	0.816
		SAL	*0.30±0.29*	0.69±1.89	0.374
		SER	**5.70±5.12**	4.04±4.37	0.006*
	SUM HDHAs	GCF	**0.71±0.45**	0.36±0.16	0.020*
		SAL	**0.23±0.18**	0.20±0.15	0.481
		SER	**13.0±14.8**	2.22±1.81	0.002*
**HEPE or HDHA**/**HETE ratios**	HEPE/HETE ratio	GCF	*0.83±1.16*	1.54±1.35	0.029*
		SAL	*0.13±0.18*	0.45±0.90	0.128
		SER	*0.03±0.01*	0.04±0.03	0.202
	HDHA/HETE ratio	GCF	*0.28±0.12*	0.45±0.17	0.005*
		SAL	*0.10±0.13*	0.16±0.18	0.309
		SER	**0.05±0.04**	0.04±0.02	0.809
**LOX**/**COX pathways**	5-LOX pathway	GCF	**5.3±3.1**	3.6±4.5	0.267
		SAL	**1.8±1.4**	0.8±0.5	0.010*
		SER	**35.6±46.7**	4.3±2.4	0.009*
	12-LOX pathway	GCF	**11.2±9.1**	6.9±3.3	0.129
		SAL	**6.2±5.2**	5.3±4.9	0.582
		SER	**286±225**	82.9±81.4	0.001*
	15-LOX pathway	GCF	**32.6±28.9**	19.9±11.2	0.166
		SAL	*12.5±12.2*	15.2±20.3	0.619
		SER	**23.2±16.3**	7.9±4.8	0.001*
	COX pathway	GCF	**5.8±2.7**	4.1±1.9	0.037*
		SAL	**4.5±10.5**	1.3±1.7	0.218
		SER	**212±153**	137±190	0.207

Ratios of mono-hydroxylated PUFA metabolites or PUFAs vs. PUFAs; sums of mono-hydroxylated PUFA-metabolites of AA, EPA and DHA and their calculated ratios; summarised metabolites originating from 5-, 12-, 15-LOX and COX metabolic pathways are shown. Data are presented in mean ± sd. * significant different (P<0.05, Mann Whitney test). Numbers in **bold** indicates increased values while *italics* indicates reduced values of AgP patients vs. healthy controls.

The sum of 5-, 12-, 15-LOX and COX pathway originating metabolites were mainly upregulated in serum, GCF and saliva samples from AgP compared with healthy controls (Table 4).

## Discussion

To draw a general picture of the contribution of bioactive lipid mediators in periodontal health and disease, lipid mediators were profiled using the HPLC-ESI-MS-MS methodology for serum, saliva and GCF samples from AgP patients and healthy controls. The precise concentration of PUFAs, PUFA metabolites and further lipid mediators determined using lipidomic technology leads to a deeper understanding of the role of lipid mediators in the mechanism of periodontal disease [31]. In this study, we report mainly elevated levels of n6- and n3-PUFAs as well as various eicosanoids and docosanoids in serum, GCF and saliva of AgP patients compared with the healthy controls. On the other hand, the ratio of the concentrations of precursors of pro-inflammatory or pro-resolving lipid mediators from n3-PUFAs vs. concentrations of n6-PUFAs (DHA and/or EPA)/AA were lower in GCF samples of AgP patients compared with healthy controls, which was the main novel finding in the present study.

EPA and DHA concentrations were significantly higher in AgP patients when compared with the healthy controls. Figueredo et al. [32] also reported of significantly higher levels of EPA and DHA in serum of patients with generalized chronic periodontitis compared with that of patients with gingivitis. Whereas there were significantly lower EPA/AA and DHA/AA ratios in GCF of the AgP patients compared to healthy controls, this was not the case in serum and saliva samples in which approximately similar ratios in AgP patients and controls were demonstrated. In a previous study it was reported that in periodontitis patients with bone loss, the serum levels of fatty acids from the n6-PUFA pathways, which were obtained after saponification, were higher than in healthy individuals, whereas the opposite was observed with fatty acids of the n3-PUFA pathway [22]. The bone loss was linked to an imbalance between the two pathways with mainly an increase in AA and a decrease in EPA and DHA.

Recently the active resolution of inflammation was recognized as a process driven by lipoxins originating from AA and novel compounds from EPA and DHA known as resolvins, which have a potential to dampen or resolve the damaging aspects of the inflammatory response. LXA_4_ is an endogenous lipid mediator which plays an important role in the local resolution processes [7], [33]. In the present study, we found LXA_4_ concentration to be increased in patients compared with healthy controls when measured by HPLC-ESI-MS-MS, but this was significant only in serum samples. LXA_4_ in GCF was also determined by ELISA. Using this technique, LXA_4_ was identified at slightly higher concentrations in healthy controls (*P* = 0.049), but the difference was minor. The pro-resolution lipid mediators such as RvD1, MaR and PD1 were detected either at very low concentrations or were below the detection limit. This can be explained either by their instability during sample collection and storage or simply by very low and undetectable endogenous concentrations with current analytical technologies [34], [35]. Recently, the “Serum Metabolome Project” identified 65 lipids by LC-MS [36]. RvE1 and RvD1 were among the lipid mediators identified in plasma of systemically healthy individuals with unknown periodontal status. Whereas these molecules have regulator functions in inflammation, the importance of circulating levels of these molecules is debated [36].

In the present study, RvD1, RvD2 and PD1 were determined in serum samples of AgP patients and healthy controls, with comparable RvD1 level. Recently, RvD1 and RvD2 were detected in the serum samples of healthy individuals having oral n3-fatty acid supplementation, while PD1 was below the detection limit [37]. Notably, in the present study, there was no n3-PUFAs supplementation of AgP patients or healthy controls. The precursor of RvE1 18-HEPE may represent the EPA-derived resolving capacity [37].

AA was present in higher concentrations in GCF samples of AgP patients than in controls. The main AA-derived pro-inflammatory lipid mediators are prostaglandins and leukotrienes. They are important biological mediators involved in the inflammatory response, as well as in regulation of bone formation and bone resorption. PGE2 has been proposed as a measure of disease status in GCF [32]. In our study, PGE2 was present at higher concentrations in GCF samples of AgP patients than in controls, which is in line with a previous study in which PGE2 concentration was significantly increased in GCF during the active phase of the disease [14]. Relatively small differences in PGE_2_ levels (ng/ml) were observed when comparing AgP patients with healthy controls in the current analysis. The mean PGE_2_ concentration in GCF was 2.2 ng/ml in AgP patients and 0.7 ng/ml in healthy controls as determined by LC-MS.

In the present study, the concentrations of the mono-hydroxylated PUFA-metabolites; 5- and 12- HETE were mainly found in higher levels in GCF and serum, while 15-HETE was found in significantly higher levels in all samples of the AgP patients. A positive association with high levels of HETEs has also been reported in other types of disease with an inflammatory component [38], [39]. Here we report that LTB4 was markedly increased in saliva samples of AgP patients, and its potential as an important marker for diagnosing periodontal disease activity should be further investigated. High concentrations of PGs, LTB4 and cysteinyl-leukotrienes have been reported to promote the accumulation of inflammatory cells such as polymorphonuclear leukocytes (PMNs) by increasing the blood flow and vascular permeability [40].

While initial fluid accumulation in the periodontal pocket seems to represent a transudate of interstitial fluid produced by an osmotic gradient leading to similar concentrations of for instance proteins in GCF and interstitial fluid, GCF is regarded as a serum exudate during inflammatory processes, with similar protein concentrations as in serum. This may also apply to the similarities in concentrations of lipid molecules in GCF and serum. Based on these findings, and with the techniques used in this study, GCF seems to be the most reliable biological fluid to reflect the local destruction (reviewed by Griffiths [41]).

The PMNs underlying the junctional epithelium in the periodontium represent the first line of defense against periodontal pathogens [42]. Transmigration of PMNs across epithelial surfaces represents a shared phenomenon among inflammatory mucosal conditions. In order to be recruited to the site of the battle, the cells depend on chemotactic signals [43]. Hepoxilin A_3_ (HXA_3_) has been shown to be a potent chemoattractant for PMNs. In our study, the concentration of HXA_3_ in GCF was not significant different between patients and controls. In serum, however, the concentration of HXA_3_ was higher in patients compared to controls (*P* = 0.005).

A balanced ratio between n3- and n6-PUFAs is suggested by World Health Organization (WHO) to decrease risk of coronary heart disease (WHO 1995). Recently, resolution actions in periodontal disease have been credited to products from the n3-PUFAs [30]. The finding in the present study further demonstrated a possible effect of n3- to n6-PUFAs ratios in AgP patients. Future studies are needed to confirm the effect of pro-resolution vs. pro-inflammatory imbalance in different types of periodontal diseases.

## Conclusions

In the present study, we report on generally elevated levels of eicosanoids and docosanoids and various n6- and n3-PUFAs in the GCF, saliva and serum of AgP patients compared with healthy controls. In addition, there were significantly increased concentrations of PGE_2_ in GCF of AgP patients compared with healthy controls determined via two independent methodological approaches. We demonstrated a lower ratio of precursors of pro-resolution to pro-inflammatory lipid mediators in the GCF of AgP patients compared with healthy controls. GCF seemed to be the most reliable biological fluid for the assessment of the periodontal condition. Findings from this study suggest that the ratio of the precursor of pro-resolution vs. pro-inflammatory derivatives might be appreciated as markers of local destruction in aggressive periodontitis. Further studies focussing on various markers for diseases severity in bigger cohorts of patients and correlating these values with various lipid markers and ratios are in progress.

## Materials and Methods

### Study population

Nineteen AgP patients (4 males and 15 females) and 19 controls (7 males and 12 females) were recruited consecutively from patients seeking treatment at the University of Science and Technology (UST), Omdurman, Khartoum, Sudan, from December 2008 to July 2009. To be included in the study, bleeding on probing (BOP), probing pocket depth (PPD) and clinical attachment level (CAL) ≥5 mm had to be present at least at one central incisor and one first molar. The patients were all <35 years (13–35 years; mean 23.4±6.4, median 23.5). The number of teeth in each patient was from 21 and 28. The diagnosis was confirmed by radiographs in addition to a full-mouth clinical examination performed by the same dentist. Before the examinations, intra-individual calibration of the examiner was undertaken. Medical history was recorded for each patient, and subjects who had received antibiotics or periodontal treatment within the last three months before the examination, or were pregnant or had any systemic disease that could affect the progression of periodontal disease, were excluded. The 19 control subjects were systemically healthy employees and students at UST, with PPD ≤3 mm and CAL ≤2 mm for all teeth, and in most cases all teeth were present.

### Ethics Statement

Ethical approval was obtained from the Research Ethics Committee at UST, Omdurman, Sudan, and the Regional Committee for Medical Research Ethics (REK) Western Norway (REK 177.04) (Bio bank no. 2355). Each subject signed an informed consent written in their native language (Arabic) prior to participation. On behalf of the young participants involved in the study, the informed consents were assigned by their guardians, all in accordance with the policy of REK, University of Bergen, and UST.

### Collection of biological materials

#### Saliva

Patients and controls were asked to rinse the mouth with water before saliva was collected in a 10 ml sterile plastic tube. The unstimulated whole saliva (UWS) samples were processed the same day by centrifuging for 10 min at 3000×g (SUPERFIT centrifuge, Mumbai, India). Each supernatant was dispersed into 4 tubes and stored in liquid nitrogen.

#### Blood

Peripheral blood was collected in vacutainer tubes; for serum. The whole tubes were preserved at bench-side for not more than two hours, and centrifuged for 10 min at 3000×g (SUPERFIT centrifuge, Mumbai|, India). The tubes with serum were then stored in liquid nitrogen.

#### Gingival crevicular fluid

A perio-paper (PERIOPAPER® Gingival Fluid Collection Strips, Oraflow Inc, New York, USA) was used for collection of GCF from the mesio-buccal site of posterior teeth (mean pocket depth of sampled teeth was 5.9 mm). Prior to the GCF collection, the area was isolated with cotton rolls and exposed to a gentle air stream for five seconds. Then the perio-paper was placed subgingivally for 10 seconds, before being removed and stored in an empty tube in liquid nitrogen.

### HPLC-ESI-MS-MS Analysis of free fatty acids, eicosanoids and docosanoids in GCF, saliva and serum

The previously described procedure for the HPLC-ESI-MS-MS method was followed with minor modifications [44], but including complete quantification of the detected lipid derivatives.

#### Sample preparation

The whole analytical sample preparation procedure is based on an established method used for retinoid quantification. Saliva samples were much lower in analyte concentration and were dried in the Eppendorf concentrator 5301 (Eppendorf, Germany) at 45°C from 1.5 ml to 50 μl prior to extraction. In summary, 50 μl of serum, saliva extract or GCF and 150 μl acetonitrile were shaken for 3 min, the precipitated protein was centrifuged at 13.000 rpm, 4°C for 6 min, 130 μL of the resulting supernatant was spiked with 10 μl isotope supernatant mix, evaporated in Eppendorf reaction vials with an Eppendorf concentrator at 30°C for ∼60 min until the sample volume was ∼10 µl. The Eppendorf concentrator was vented with argon to prevent degradation of eicosanoids and docosanoids. The dried extract was resuspended with approximately 25 μl of HPLC solvent A [64.3% water (water, Chromasolv Plus from Sigma-Aldrich, Hungary) and 35.5% acetonitrile (Merck KGaA, Germany) and 0.2% formic acid (Fluka, Hungary)] to yield 35 μl, then vortexed (15 sec), shaken (3 min) and transferred into micro injection inserts vials (Waters, Hungary). These glass vials with the 35 μl extract were transferred into brown screw top vials with PTFE/silicone septa sample (Waters, Hungary) and placed in the pre-cooled (15°C) autosampler of the Waters 2695XE separation module.

#### Chromatographic system

The HPLC system consisted of a Waters 2695XE separation module (Waters, Hungary) including a gradient pump, autosampler, degasser and a heated column compartment. A MS-MS detector with an ESI ionizing option was used (Micromass Quattro Ultima PT from Waters, UK; a gift from Biosystems Int., France) as a detector. The system was controlled via the MassLynx software (Waters, Hungary).

#### HPLC conditions

The eluents were degassed in the Waters 2695XE separation module prior to mixing, then passed through an in-line filter (1–2 μm; Knauer, Germany) before reaching the analytical column (LiChroCART, 125 X 2 mm; Superspher 100, RP-18, endcapped) from Merck KgaA (Germany) embedded in the column compartment. A multilinear gradient was formed from solvent A (see above) and solvent B (methanol; Merck KGaA Germany). The gradient consisted of the following steps: 0.0 min 20% B, 3.0 min 20%B, 5.0 min 60%B, 15.0 min 100%B, 15.9 min 100%B and 16.0 min 5%B. The flow rate was adjusted to 0.4 ml/min and the column was heated to 40°C. From the same biological extract, 10 μl for each HPLC analysis was used. This step was performed twice using the same HPLC conditions and two different MS-MS analysis options for better resolution and quantification of the various analytes.

#### MS options

The Micromass Quattro Ultima PT was controlled via the MassLynx software. Argon with an inlet pressure of 0.8 bar was used. ESI (electro spray ionization source, Waters, Hungary) was vented by nitrogen continuously produced by the nitrogen generator (Peak Scientific NM30 Nitrogen generator) including compressor (Waters, Hungary) with the inlet flow set at 3.6 e-3 mbar.

Multiple reaction monitoring settings: ESI, with a negative ESI – setting, was performed with the HPLC eluent following the ion source temperature of 85°C. The desolvation gas flow was 780 l/h, the desolvation temperature was 400°C, the cone gas flow was 10 l/h, the capillary current was 3 μA, and the cone voltage was 50 V. Aperture voltage was set at 0 V and the RF lens voltage was set at 35 V (for 1) and 0.2 V (for 2). The analyzer settings were LM1 resolution 14.5; HM1 resolution 14.5; Ion energy 1 0.7; entrance –1; collision 0 (collision parameters are set for each substance at the MS – method parameters); exit 2; LM2 resolution 14.5; HM2 resolution 14.5; Ion energy 25.0 and a multiplier energy of 650 V.

Multiple reaction monitoring settings for PUFA, eicosanoids/docosanoids semi-quantification: Method A from 0.0 – 9.0 min for PGF_2_ 349.0 ->192.7, collision energy 22 eV; PD1 and PD1 isomers like PDX 359,0 ->153.3, collision energy 17 eV; TXB_2_ 369.0 ->195.0, collision energy 13 eV, PGE_3_ 349,0 ->233,0, collision energy 17 eV, LXA_4_/LXB_4_ 351,0 ->115.0, collision energy 12 eV, 8iPGF_3_ 351.0 ->193.0, collision energy 22 eV, 20-COOH-LTB_4_ 365.0 ->195.0, collision energy 13 eV, RvD1, RvD2 375.0 ->141.3, collision energy 13 eV; RvE1 375.1 ->141.3, collision energy 13 eV; from 9.0–12.5 min for 13-HODE 294.7 ->170.7, collision energy 16 eV; 9-HODE 294.7 ->194.7, collision energy 16 eV; 5-HEPE 317.0 ->115.0, collision energy 17 eV; 12-HEPE 317.0 ->179.0, collision energy 17 eV; 15-HEPE 317.0 ->219.0, collision energy 17 eV, LTC_4_ 623.9 ->272.0, collision energy 14 eV from 12.5–16.0 min for LA 279.3 ->279.0, collision energy 10 eV; 8-HEPE 317.0 ->255.0, collision energy 17 eV, 18-HEPE 317.0 ->259.0, collision energy 17 eV; 5-oxoETE, 12-oxoETE and 15-oxoETE 317.0 ->273.0, collision energy 17 eV, 20-HETE 319.0 >245.0, collision energy 10 eV; LTC_4_ 623.9 ->272.0, collision energy 14 eV; LTE_4_ 438.0 ->333.0, collision energy 13 eV from 12.5–16.0 min for LA 279.3 ->59.2, collision energy 25 eV; EPA 301.0 ->203.2, collision energy 12 eV; AA 303.0 ->259.3, collision energy 11 eV, DHA 327.1 ->29.3, collision energy 14 eV.

Method B from 0.0–9.8 min for PGE_2_, d15d12PGD_2_, PGD_2_, d15d12PGJ_2_, PGJ_2_ 315.0 ->271.3, collision energy 13 eV; LTB_5_ 333.0 ->195.0, collision energy 13 eV; LTB_4_ 335.0 ->195.0, collision energy 13 eV; RvE_1_ 349.1 ->195.3, collision energy 13 eV; HXA_3_, HXB_3_, 20-COOH-AA 335.0 ->273.3, collision energy 13 eV; LXA_5_ 349.0 ->115.0, collision energy 12 eV; 20-OH-LTB_4_ 351.0 ->195.0, collision energy 13 eV; MaR 359.0 ->250.0, collision energy 13 eV; from 9.8–12.5 min for 5-HETE 318.7 ->115.0, collision energy 14 eV; 8-HETE 319.0 ->155.0, collision energy 14 eV; 11-HETE 319.0 ->167.0, collision energy 14 eV; 12-HETE 319.0 ->179.0, collision energy 14 eV; 15-HETE 319.0 ->218.9, collision energy 11 eV; 4-HDHA 343.0 ->101.0, collision energy 10 eV; 10-HDHA 343.0 ->181.0, collision energy 10 eV; 14-HDHA 343.0 ->205.0, collision energy 10 eV; 17-HDHA 343.0 ->245.0, collision energy 14 eV, 20-HDHA 343.0 ->285.0, collision energy 10 eV; 13-oxoODE 293.0 ->249.0, collision energy 17 eV and from 12.5–16.0 min for LA 279.3 ->59.2, collision energy 25 eV; EPA 301.0 ->203.2, collision energy 12 eV, AA 303.0 ->259.3, collision energy 11 eV, DHA 327.1 ->229.3, collision energy 14 eV.

#### Standard solutions

Stock solutions of the PUFAs, eicosanoids and docosanoids were prepared by dissolving the solutions obtained from Cayman-Chemicals (Estonia), BioMol International (Kastel-Med KFT, Budapest, H), Sigma-Aldrich (Budapest H), Larodan Lipids (Malmö, Sweden) and Dr. Charles Serhan (Harvard, USA) with methanol to yield a final concentration of 10 μg/ml. All stock solutions were stored in darkness at −80°C. The reference PUFAs, eicosanoids and docosanoids were used for the assay validation.

#### Quantification

Individual eicosanoids and docosanoids were quantified based on the determination of the “Area Under the Curve” (AUC) and compared with the AUC of standard compounds. To ensure optimal extraction isotope labelled standard compounds were used. This analytical procedure was established for liquids and tissue analysis [45].

### ELISA

#### Levels of PGE_2_ and LXA_4_ in GCF

Perio-papers saturated with GCF from AgP patients and controls were immersed in 12 mM (pH 7.6) Tris buffer. The lipids recovered from the GCF samples were diluted in the assay buffer of the ELISA kit; 10 times for the analysis of PGE_2_ and 5 times for the LXA_4._ The PGE_2_ and LXA_4_ were quantified by the competitive ELISA (Neogen Corporation, Nandino Blvd., Lexington KY, USA) according to instructions provided by the manufacturer. All GCF samples were run in duplicate and measured by FLUOstar OPTIMA, and data were reported in ng/ml.

### Statistical analysis

Values which were determined under the detection limit were replaced with values which present half of our quantification limit: 0.02 ng/ml for saliva and 0.05 ng/ml for GCF and serum. The difference in significance between AgP and healthy controls was determined by Predictive Analytics Software (PASW) version 18, using the Mann Whitney test. *P* values <0.05 were considered statistically significant.
